# Artificial intelligence for dementia—Applied models and digital health

**DOI:** 10.1002/alz.13391

**Published:** 2023-07-26

**Authors:** Donald M. Lyall, Andrey Kormilitzin, Claire Lancaster, Jose Sousa, Fanny Petermann‐Rocha, Christopher Buckley, Eric L. Harshfield, Matthew H. Iveson, Christopher R. Madan, Ríona McArdle, Danielle Newby, Vasiliki Orgeta, Eugene Tang, Stefano Tamburin, Lokendra S. Thakur, Ilianna Lourida, The Deep Dementia Phenotyping (DEMON) Network, David J. Llewellyn, Janice M. Ranson

**Affiliations:** ^1^ School of Health and Wellbeing College of Medical and Veterinary Sciences, University of Glasgow Glasgow UK; ^2^ Department of Psychiatry University of Oxford Oxford UK; ^3^ School of Psychology University of Sussex Brighton UK; ^4^ Personal Health Data Science SANO‐Centre for Computational Personalised Medicine Krakow Poland; ^5^ Faculty of Medicine Health and Life Science, Queen's University Belfast Belfast UK; ^6^ Centro de Investigación Biomédica Facultad de Medicina, Universidad Diego Portales Santiago Chile; ^7^ Department of Sport Exercise and Rehabilitation, Northumbria University Newcastle upon Tyne UK; ^8^ Stroke Research Group, Department of Clinical Neurosciences University of Cambridge Cambridge UK; ^9^ Centre for Clinical Brain Sciences University of Edinburgh Edinburgh UK; ^10^ School of Psychology University of Nottingham Nottingham UK; ^11^ Translational and Clinical Research Institute Faculty of Medical Sciences, Newcastle University Newcastle upon Tyne UK; ^12^ Division of Psychiatry University College London London UK; ^13^ Department of Neurosciences Biomedicine and Movement Sciences, University of Verona Verona Italy; ^14^ Broad Institute of MIT and Harvard Cambridge Massachusetts USA; ^15^ University of Exeter Medical School Exeter UK; ^16^ Alan Turing Institute London UK

**Keywords:** AI, applied models, artificial intelligence, dementia, digital health, machine learning, ML

## Abstract

**INTRODUCTION:**

The use of applied modeling in dementia risk prediction, diagnosis, and prognostics will have substantial public health benefits, particularly as “deep phenotyping” cohorts with multi‐omics health data become available.

**METHODS:**

This narrative review synthesizes understanding of applied models and digital health technologies, in terms of dementia risk prediction, diagnostic discrimination, prognosis, and progression. Machine learning approaches show evidence of improved predictive power compared to standard clinical risk scores in predicting dementia, and the potential to decompose large numbers of variables into relatively few critical predictors.

**RESULTS:**

This review focuses on key areas of emerging promise including: emphasis on easier, more transparent data sharing and cohort access; integration of high‐throughput biomarker and electronic health record data into modeling; and progressing beyond the primary prediction of dementia to secondary outcomes, for example, treatment response and physical health.

**DISCUSSION:**

Such approaches will benefit also from improvements in remote data measurement, whether cognitive (e.g., online), or naturalistic (e.g., watch‐based accelerometry).

## 1 INTRODUCTION

Different sources of data have different strengths.^1^ Integrating, maximizing, and harmonizing such sources with their offsetting, complementary characteristics, is a major challenge and opportunity of our age and field—good science requires multiple lines of evidence from different sources of data.^2^ It is a complex challenge to develop ideal models to maximize these resources. Digital health tools such as wearables are a potentially highly informative area for dementia risk reduction, improving diagnosis and prognosis research as they provide objective measurement of data that was previously mostly subjective (e.g., physical activity, sleep quality), plus incidental measurement at scale. This results in more detailed information with fewer biases and potentially at larger scales.^3^ Although the analysis and derivation of usable information from raw data are complex, this is a key area of progress in dementia research. This narrative review article will discuss the current state of applied models and digital health regarding dementia risk prediction, diagnostic models (e.g., discriminating mixed dementias), prognostics/progression, and potential future applications, with a predominant focus on emerging data sources and their integration.

We organize the paper around three modeling problems: dementia risk prediction, diagnosis, and prognosis. In the risk prediction problem, the goal is to predict the risk of future dementia among people who do not currently have dementia. The goal of a diagnostic model is to detect, as early as possible, when a person develops dementia. The goal of a prognostic model is to predict how dementia will progress among patients who already have dementia. We discuss how these problems interact with emerging technologies and what that means for modeling, and finally discuss future prospects and fundamental limitations.

This review is one of a series of eight articles in a special issue on “Artificial Intelligence for Alzheimer's Disease and Related Dementias” published in *Alzheimer's & Dementia*. Together, this series provides a comprehensive overview of current applications of artificial intelligence (AI) to dementia, and future opportunities for innovation to accelerate research. Each review focuses on a different area of dementia research, including experimental models, drug discovery and trials optimization, genetics and omics, biomarkers, neuroimaging, prevention, applied models and digital health, and methods optimization.

## 2 RISK PREDICTION

Looking at long‐term dementia risk and the preclinical/asymptomatic stage: How early can we detect aspects of dementia? What types of data are likely to be useful?

### 2.1 Multi‐omic integration with machine learning (ML)

Machine learning (ML) is the use of automated algorithms to develop models from data, which can predict an outcome with the best possible accuracy. “Training” datasets (typically 10%–20% of the study total) are used to develop a model, and “test” datasets are used to test its accuracy. A 2021 systematic review of 64 papers reported that there was wide variety across studies which used ML to predict dementia. Reports varied in sample sizes (including training vs. test sets), the sets of variables explored and identified (e.g., genetic mutations were reported in only 38% of studies), and exact ML methods used (e.g., decision trees, Bayesian networks, neural networks). Dementia risk prediction models often incorporate a diverse range of variables in combination, to generate a measure of an individual's risk of developing future illness.^4^, ^5^ A commonly cited example is the Cardiovascular Risk Factors, Aging, and Incidence of Dementia (CAIDE) dementia risk score, which incorporates demographic (age, education, sex), health (hypertension, body mass index, physical activity), and blood marker (cholesterol) variables to predict, using logistic regression, the mid‐life risk of dementia.^6^ Biomarkers such as plasma phospho‐tau can predict Alzheimer's disease (AD) on their own. However, using such biomarkers in combination with other markers, for example, cognitive (memory, executive function) and genetic (e.g., apolipoprotein E [*APOE*]) data, can increase the accuracy of a logistic regression‐based classification model in a cohort of participants with subjective cognitive decline and mild cognitive impairment (MCI).^7^ As well as the overall accuracy of the model, the combination of variables used is likely to be influenced by ease of accessing the variables, cost effectiveness, and where the model is likely to be used, for example, clinical versus research settings.

The integration of multiple phenotypes (e.g., biomarkers, anthropometric, neurocognitive) with ML and data science approaches has great potential in understanding markers of dementia, for example, the use of magnetic resonance imaging (MRI) to identify brain biomarkers of MCI and dementia.^8^ Using various classification models, ranging from linear logistic regression to non‐linear gradient boosting trees, blood proteomic biomarkers can predict cognitive impairment^9^ including potentially as an efficient tool for pre‐screening and recruitment for clinical trials.^10^

### 2.2 Cohorts

Life‐course predictors of dementia risk and onset are often addressed using longitudinal studies of aging such as the Health and Retirement Study,^11^ Framingham Heart Study,^12^ or the Alzheimer's Disease Neuroimaging Initiative (ADNI^13^). Longitudinal cohort studies have had success in identifying factors from across the life course that predict increased dementia risk, both proximal factors—such as depressive symptoms^14^—and factors from early life such as low educational attainment.^15^ However, many longitudinal cohort studies suffer from problems with representativeness. For example, the UK Biobank is biased toward older, less deprived, and more physically and psychologically healthier individuals.^16^, ^17^ Participation bias therefore is a fundamental limitation to non‐routine data, which requires opt‐in.

**RESEARCH IN CONTEXT**

1. **Systematic Review**: This narrative review article examined literature pertaining to the application of applied models (e.g., machine learning) and digital health (e.g., remote testing, objective sensors) in dementia research.
2. **Interpretation**: Our synthesis suggests substantial promise in applied modeling and digital health to enhance our ability to measure dementia risk, progression, and potential treatment response. Multiple challenges exist for researchers however, for example, in the convenient access and analysis of anonymized secondary data.
3. **Future Directions**: We make specific recommendations for the field moving forward. These include emphasis on transparent and reproducible analyses; incorporation of multiple ‐omics into modeling; movement toward secondary outcomes like progression from mild cognitive impairment to dementia; integration of novel phenotyping from electronic health records and neurolinguistic programming; the use of and development of bespoke tools for including naturalistic “real‐world” data in modeling.

Linkage of routinely collected data has allowed the development of large, population‐wide studies of dementia that are more representative than traditional longitudinal studies and can provide more accurate prevalence and risk estimates.^18^ Recent advances such as the Secure Anonymised Information Linkage (SAIL) databank in Wales,^19^ which provides remote and secure access to national, participant‐level social and health‐care data, have made it easier for researchers to design case–control studies^20^ and to link large amounts of data—particularly health data—to create population‐wide e‐cohorts. Such studies have shown the importance of risk factors such as mid‐life psychiatric disorders.^20^

There is potential utility in optimizing ML for secondary and potentially tertiary care, including the longitudinal conversion to dementia. James et al.^21^ for example showed that gradient boosted trees achieved 92% accuracy in predicting incident dementia (*n* = 32,573 participants across 2 years). Critically, multiple ML approaches were significantly more accurate than existing models such as CAIDE and the Brief Dementia Screening Indicator (BDSI) (≈80% vs. 92%). This to some extent probably reflects that CAIDE/BDSI were designed for much longer follow‐up than 2 years. Classification of dementia subtype was 40% to 50% accurate across multiple ML methods, for example, random forest (RF), support vector machine (SVM), and so on, suggesting room for improvement in the future. A difficulty in complex modeling using a large number of variables is that a substantial amount of variance is probably captured by relatively known variables, like age, female sex, and *APOE* ε4 genotype.^22^ There are few to no examples of hypothesis‐free testing generating a truly novel modifiable (phenotypic) risk factor.

Linkage of routinely collected data allows examination of environmental risk factors, such as aluminum and fluoride in drinking water^23^ and air pollution.^24^ Li et al.^25^ demonstrated the potential of integrating UK Biobank data^26^ with secondary health electronic medical records. Such linkage opens unparalleled opportunities for epidemiological analyses and longitudinal mental health and cognitive studies by leveraging rich multimodal genetic, environmental, and imaging data from the UK Biobank with precise observational medical history.

While population data linkage provides a low‐cost, representative way of following large samples longitudinally, researchers often have no control over the timing and frequency of observations, and some types of data are not readily available. The accuracy of records in terms of dementia coding also varies among health‐care settings.^27^ This makes data linkage on its own not necessarily suitable for tracking cognitive decline; (normative) cognitive test performance is rarely recorded and is measured infrequently compared to traditional longitudinal studies. Similarly, for examining certain biological mechanisms, key data for examining small‐scale changes in biology or behavior during preclinical and prodromal phases are not recorded. More fundamentally, routinely collected data are generally not created for the purpose of research, and coding schemes in particular may not be aligned with research interests.^28^ Instead, data linkage can be used to enhance or complement more traditional longitudinal studies.^25^ ML therefore has significant potential to inform conversion to AD, including decomposing large numbers of variables to relatively few both via dimensionality reduction (e.g., principal components analysis, Uniform Manifold Approximation and Projection) and on the basis of feature importance (i.e., individual contributions to the model).

### 2.3 Digital measurement

Cognitive impairment based on neuropsychological testing may emerge relatively late in the development of AD.^29^ Digital assessments may promote improved sensitivity compared to brief screening tools in several ways. First, while there still is a tendency to translate “in‐clinic” tools for a remote interface, technology presents an opportunity to develop new paradigms, designed to assess cognitive processes linked to early pathological processes. For example, the Mezurio app includes several tasks^30^ that focus on perirhinal, entorhinal, and hippocampal processes—sub‐regions vulnerable to early neuropathological tau deposition. Virtual reality spatial navigation tasks assess entorhinal function in an ecologically valid setting.^31^ However, data loss can occur due to technical issues/participant error which means more can be done to build scalable, accessible tests. Second, digital tools present the opportunity for remote, repeat assessment. This allows researchers to measure aspects of cognition that cannot easily be measured in face‐to‐face settings. For example, Mezurio measures memory for object–direction pairings over variable delays of up to 2 weeks (including Gallery Game^30^). Multiple testing platforms exist, for example, NIH Toolbox, Cogstate.^32^ Finally, the sensors in digital devices allow for a range of signatures for example, voice, connected speech, fine‐motor control, and typing speed, to be collected alongside predominant response time and accuracy.^33^ Recent ML improvements to architecture, such as multimodal transformers,^34^ can leverage diverse input streams (i.e., varying data types/formats) to learn signatures of cognitive impairment and use them for predictive and inferential analytical tasks. Speech markers are proving increasingly useful for detecting variables that may demarcate future or current AD;^35^ specifically, speech transcripts have been transformed into linguistic features (e.g., filled and unfilled pauses, repetitions, and semantic units) and subsequently used with mixed‐effects linear models to predict AD status. An ensemble ML model comprising SVM and the k‐nearest neighbors, using as input the paralinguistic speech features (e.g., pitch, volume, speech rate) augmented with memory tests, has achieved 97.2% accuracy of distinguishing participants at high and low risk of dementia.^36^ The transfer learning paradigm with further domain adaption, allowed development and validation of an ensemble ML model comprising a combination of RF and gradient boosting machine (GBM) algorithms, achieving accuracy of 87%, specificity of 99%, and sensitivity of 76% in predicting individuals’ risk of developing dementia.^37^

With greater refinement and carefully applied data science techniques, researchers may be able to detect dementia earlier in the disease trajectory. Critically, digital tools allow for greater precision: participants are often willing to complete cognitive assessments frequently and for sustained durations.^38^ This, coupled with sensitive, targeted, sensor‐based measurement, may help us detect current signatures or predictors of future AD that have been missed in past research.

## 3 DIAGNOSTIC MODELS

To be able to distinguish dementia diagnosis from other cognitive disorders is a fundamental requirement for better care, treatment, and prognosis.

Diagnostic models of dementia have a multidimensional nature in which developments in data quality (e.g., objective measurement of previously subjective phenotypes) and modeling improvements (directly) taken together can provide synergistic improvements. ML approaches benefit extensively from advances in medical and health science data.^39^, ^40^ While multimodal integration may benefit ML there are currently two key challenges to this. First, integrating already available data sources such as multi‐modal omics (defined as any relatively objective biologic measurement), biomarkers including anthropometric, and variables being collected regularly as part of standard care. Second, incorporating relatively novel data sources such as wearables.^3^ While substantial ML research has been conducted to address the former by developing technology and concepts around federated ML to overcome data sharing and sample size constraints, little has been done to use the latter in the ML process. A more general challenge is incorporating digital technology with occasionally noisy data.^41^, ^10^

It is not necessarily the case that objective data are “superior” to self‐report or subjective data in all instances. Self‐reported cognitive decline can be more important information in the absence of premorbid data, and multiple forms of data (e.g., electronic health records [EHRs], self‐reported histories, biomarker‐based ascertainment) are complementary^42^ rather than necessarily hierarchical.

A key aim of applied modeling is to reduce the time to diagnosis and the identification of dementia.^43^ Ford et al.^44^ showed that routine, non–dementia‐specific standard clinical data could be used to identify cases 5 years before diagnosis (*N* = 93,120 participants with dementia). The primary features were neuropsychiatric, self‐care, and family history of dementia. They found that while naive Bayes modeling performed least well (area under the receiver operating characteristic curve [AUROC] = 0.68), logistic regression, SVM, neural networks, and RF (AUROC all ≈0.74) performed similarly. While this demonstrates proof‐of‐concept that ML can be used to identify dementia cases, more data could improve modeling further, including genetics, biomarkers, and imaging.^45^

A particular diagnostic problem of interest is identifying rare dementia subtypes for which less data are available. Digital technology offers a potentially easy, relatively inexpensive solution to improving the identification and differentiation of dementia subtypes. One notable example of the growing evidence for digital markers of dementia is the use of wearable technology and digital mobility outcomes for early and accurate diagnosis.^46^ Digital mobility markers such as gait and physical activity have gained interest as digital biomarkers for predicting dementia, and the ability of wearable technology to capture mobility markers through continuous remote‐monitoring methods in the real world.^46^, ^47^ Current research provides evidence that wearable‐based gait impairments (e.g., speed, variability) are associated with cognitive decline^48^ and dementia, and can differentiate between dementia subtypes.^49^ Similarly, different volumes and patterns of physical activity have been found between people with dementia or MCI versus healthy older adults, using continuous remote monitoring methods with accelerometers and multiple regression models^50^ as well as latent difference score models,^51^ with significant differences observed between non‐AD subtypes such as dementia with Lewy bodies and Parkinson's disease dementia.^49^ Given the increasing interest in remote clinical practice and diagnostic assessments, digital mobility markers may be a useful addition to the clinician's toolkit. However, research is still in the relatively early stages. Further work is required to identify the most useful digital mobility metrics that can be incorporated into classification models such as ML algorithms to strengthen diagnostic models. This includes the extension of such digital health metrics to ML modeling.

Digital mobility markers are only one example of potential digital biomarkers for aiding dementia diagnosis and will be most useful as part of a diagnostic battery.^52^ Significant research efforts are assessing the efficacy of other modalities to aid early differential diagnosis of dementia and its subtypes, including digital markers of sleep,^53^ speech processing,^54^ and cognition.^38^ International consortiums such as the Early Detection of Neurodegenerative Diseases (EDoN) Initiative are examining combinations of these digital biomarkers for the development of a digital toolkit and harnessing the power of ML, such as GBM or probabilistic multiple kernel learning methods and deep neural network–based models to detect dementia in early and prodromal stages of the disease.^52^ Once these methods have been validated against gold‐standard biomarkers such as neuroimaging and cerebrospinal fluid,^55^ they may provide clinicians an inexpensive tool with utility for early detection of dementia, including in regions with limited health‐care resources.^56^

Rare and early onset dementia, such as prion diseases, rare genetic variants of frontotemporal dementia (FTD), primary progressive aphasia (PPA), and uncommon variants of AD (e.g., posterior cortical atrophy) may be poorly represented in outpatient clinics (except in tertiary centers), as well as in studies assessing diagnostic models.^57^ Their low prevalence may result in increased negative and reduced positive predictive value, thus raising the likelihood of a false positive finding. There is evidence that using deep feed‐forward neural network with acoustic and linguistic variables may correctly subtype and classify PPA variants and behavioral variants for FTD,^33^, ^57^ with 80% accuracy, significantly outperforming common ML approaches, such as RF (58% accuracy) and SVM (45% accuracy). Future studies should explore the ability of these models to diagnose PPA versus AD and FTD, and measure the sensitivity and specificity according to the sample to increase their applicability in different types of dementia centers.

## 4 PROGNOSTIC MODELS AND MEASUREMENT OF DISEASE PROGRESSION

How can new technologies support prevention, diagnosis, and provision of care for people with dementia? Which digital biomarkers may be the most useful?

### 4.1 Variable rates of progression and clinical heterogeneity

Substantial bodies of ML research have focused on integrating brain imaging with structured and unstructured clinical data to predict disease progression; for example, these have included neurobehavioral exam scores and clinical notes, respectively.^58^ A difficulty in measurement of decline/prediction of progression in AD research is heterogeneity, which is best approached with either very large datasets or integration of multimodal data, for example, imaging, biomarkers, and demographics.^59^ Kumar et al. provide a systematic review of ML applied to AD progression (including use of regression, SVM, decision trees, Bayesian and neural networks, and natural language processing [NLP]), in particular highlighting the potential of unsupervised approaches.^58^ A major benefit of unsupervised ML includes the potential to identify novel sub‐phenotypes and distinct trajectories.^60^ Fisher et al.,^61^ for example, describe unsupervised conditional restricted Boltzmann machine (CRBM) learning approach to simulate high‐fidelity patient trajectories, showing efficacy at identifying fast versus slow progressors on synthetic (i.e., artificial) data. This approach used relatively sparse data (44 variables) as a proof‐of‐concept but shows significant promise as the study expands to broader, multimodal datasets.

Among relatively novel digital biomarkers, wearable technology such as accelerometers and inertial measurement units (IMUs) have been used to measure subtle changes in gait, sleep, and physical activity in dementia.^49^ Recent studies have shown that these markers are able to detect the unique signatures of gait and different volumes, patterns, and variability of physical activity of people through the dementia process.^46^, ^47^, ^49^ Therefore, measuring change over time in gait and physical activity may provide important information about disease progression, which may not be detected through repeated cognitive assessments due to practice effects. Additionally, they can be assessed continuously and remotely rather than only at clinical visits.^62^

Regarding physical activity, most evidence  has been hitherto derived from data based on self‐report measures.^63^ Digital biomarkers such as device‐measured physical activity (using methods like accelerometry and movement sensors), are feasible at scale and provide more objective measures of physical activity, allowing researchers to distinguish between different levels and intensities of activities.^64^ Recent studies have focused on measuring the volume of physical activity, such as step counts or time spent walking. Calculating the total volume of physical activity is an important primary outcome for populations experiencing cognitive impairment. Measuring patterns such as day‐to‐day changes and mean bout length, or variability of physical activity, allows researchers to predict changes in habitual routines associated with dementia progression.^46^ In addition to monitoring disease progression, digital markers such as gait and physical activity are likely to be key for predicting important post‐diagnostic outcomes, such as the risk of falls,^65^ and functional decline.^66^ There is additional scope for the role of physical activity (or general exposure) in green spaces.^67^

Sleep disturbances can become increasingly common during the course of dementia; sleep markers can reflect this decline and allow clinicians to adapt care provision for an individual's need. Polysomnography is the gold standard as it provides estimates of the overall sleep architecture (including non‐rapid eye movement [NREM] and rapid eye movement [REM]).^68^ Other monitoring sensor devices (e.g., actigraphy), can variously capture circadian rhythms, mood, global cognitive status, and sleep disruptions.^69^ Although actigraphy cannot determine NREM or REM, the device provides measures of sleep latency, total sleep time, wake after sleep onset, and sleep efficiency.^68^

Combined with their relative economy and accessibility, sensor devices will be very useful in future longitudinal studies in which long‐term changes in several outcomes are of particular interest (such as physical activity and sleep patterns).^49^ Digital technology, therefore, may allow clinicians to gain a better understanding of disease progression and monitor populations at high risk of adverse outcomes. Objective measurement of previously subjective metrics (diet, activity, sleep) could extend to broader phenotypes including diet, heart‐rate variability/stress, and smoking intensity. Future interventions to negate adverse events through digital technology have great potential in improving personalized post‐diagnostic care for people with dementia.

### 4.2 Digital technologies in real‐life environments

Most research to date has focused on the applicability and use of embedded environmental sensors or wearables in real‐life home environments to monitor everyday mobility and function.^46^ Several small‐scale studies have shown that global positioning system (GPS)–enabled technologies applied to monitor ‘out‐of‐home’’ activities and mobility in early AD are generally feasible and valid, comparable to more traditional paper and pencil measures.^70^ The potential usability of these devices is further supported by data that monitoring activities of daily living using GPS‐enabled technologies can reliably distinguish between mild to moderate stages of AD.^71^ In addition, emerging new perspectives such as those focusing on the measurement of “life space behavior” (i.e., GPS‐tracked daily routines) may be particularly valuable in informing disease progression in mild AD.^72^ Preliminary data suggest that increasing “life space behavior” may reduce symptoms related to inactivity such as apathy and maintain physical health in mild dementia.^72^ However, it should be noted that significant work is required to validate digital technologies for clinical use in real‐world environments.^46^ Research needs to move from pilot studies to large‐scale longitudinal multi‐disciplinary studies to best understand, synthesize, and standardize protocols, data outcomes, and interpretation of findings. Algorithms applied by digital technology need to account for variances in real‐world environments, such as location or constrained settings, and validation against “gold standards” (e.g., imaging markers) must show “true” clinical efficacy. The perspectives of clinicians, patients, and families should be integrated into developing digital health‐care innovative interventions to ensure feasibility and usability. Researchers should also work in tandem with regulatory bodies to ensure outputs meet the requirements of a clinical tool.

### 4.3 Treatment response in trials

Despite the potential usability of the above‐described devices providing ecologically valid feedback, most studies to date remain small and observational in nature, limited by small sample sizes.^56^ Research growth in this area is hindered by the lack of international guidelines on which devices are more likely to be useful in monitoring disease progression in AD and which may be more feasible as embedded clinical outcomes in trials.^73^ Interdisciplinary approaches, supplemented by rigorous co‐production and co‐design processes alongside people with dementia and key stakeholders, are key to progress in the area. Despite the relative paucity of research on which digital biomarkers have the most utility to inform disease progression in dementia, those that monitor activities of daily living using less invasive approaches are promising and require further research. Future rigorous large‐scale longitudinal studies on “home‐based real‐world evaluations” and those informed by co‐production of knowledge alongside key stakeholders will be key in translating how these approaches may offer direct patient benefit, improving pre‐ and post‐diagnostic care for people with dementia.

## 5 LOOKING TO THE FUTURE

### 5.1 What new research resources would be transformative?

#### 5.1.1 Transparency, sharing, and harmonization

The inclusion of EHR in existing large‐scale datasets is a key resource for dementia research, yet more needs to be done to facilitate the efficient, effective, and safe use of raw data. The UK government, for example, recently launched a review into how best to ensure this resource maximally benefits researchers, patients, and the health‐care sector (https://www.gov.uk/government/news/new‐review‐into‐use‐of‐health‐data‐for‐research‐and‐analysis). A fundamental scientific and ethical aim is the promotion of global health including low‐ and middle‐income countries and across all global demographics; therefore, a concerted international effort is required.^74^ There is an ethical imperative to understand population‐level diversity in disease prevalence and outcomes; additionally, there is potential mechanistic understanding to be gained from these differences. Data sharing is an increasingly common practice; however, data harmonization is important for analyzing information across multiple sources. Already we see examples of the cross‐process harmonization of EHRs (e.g., Observational Health Data Sciences and Informatics: https://www.ohdsi.org/data‐standardization/the‐common‐data‐model/), and data platforms which integrate outcomes across cohorts, for example, Dementias Platform UK (DPUK), Alzheimer's Disease Data Initiative (ADDI), and Dementias Platform Australia (DP Australia). EHRs are increasingly being incorporated into existing for‐research cohorts, such as Generation Scotland and UK Biobank. For data harmonization to progress, however, infrastructure must be further developed to include suitable data platforms, open‐access processing pipelines with adequate computing power, easy‐to‐navigate data dictionaries, and tools for characterizing complex data structures. Harmonization with improved infrastructure will not only allow “big data” analyses using traditional statistical methods, but enable AI to be applied across diverse datasets and modalities. In turn, this will allow a triangulated approach to hypothesis testing, greater generalizability, and replication via independent datasets. There is a need therefore for research to be large scale and multi‐disciplinary including (1) researchers from multiple backgrounds,^75^ (2) inclusion of patient and public involvement as standard to emphasize what “matters” to affected individuals and their families, and (3) emphasis on replicability and open scientific practices (e.g., preregistrations, shared code).

#### 5.1.2 Natural language processing for electronic health records

The prevalence of EHRs in Mental Health UK is higher than most other secondary health‐care systems; mental health National Health Service (NHS) trusts in England and Wales have been digitized with EHRs for more than a decade. The major secondary care mental health EHR platforms, such as the South London and Maudsley NHS Foundation Trust Clinical Record Interactive Search (SLaM CRIS: https://www.slam.nhs.uk/quality‐and‐research/clinical‐record‐interactive‐search‐cris/) and the Oxford‐led federated Clinical Record Interactive Search network (UK‐CRIS: http://www.awp.nhs.uk/about‐us/rd/uk‐cris) provide access to pseudonymized structured and unstructured free‐text datasets under strict safeguard measures. Advances in NLP and text mining have enabled in‐depth secondary analysis of the real‐world effectiveness of multiple aspects of clinical care. These include: the effectiveness of cholinesterase inhibitors, memantine and trazadone as dementia treatments,^76^, ^77^ the development of open‐source information extraction tools,^78^ evaluating the feasibility of using clinical records with the validated suicide risk assessment tool^79^ to extract patterns pertinent to major depressive disorders,^80^ and demonstrating the feasibility of using ML models, such as long short‐term memory recurrent neural networks, for personalized treatment recommendations in people with cognitive decline.^81^ Recent advances in transformers‐based large language models, which outperformed traditional recurrent neural networks, and the availability of large population‐level electronic textual records hold strong promise to transform and support research in dementia and^82^ neurodegenerative disorders.

#### 5.1.3 E‐cohorts and remote testing

Increasing numbers of internet‐based registries will accelerate recruitment to dementia trials. Incorporating remote cognitive and lifestyle assessment, plus clinical history, will help match the appropriate people with each clinical trial. The Brain Health Registry^83^ has longitudinally collected phenotypic data from the general public, resulting in the recruitment of ≈19,000 participants to clinical trials. The value of these registries can be further boosted by acquisition of easy‐to‐collect biological samples, for example GeneMatch has profiled nearly 80,000 volunteers for *APOE* ε4 “risk” genotype, for the purpose of recruitment to clinical research.^84^ Registries can work with existing cohorts—for example, DPUK Clinical Studies and Great Minds register seeks to recruit up to 3 million participants involved in > 40 cohorts to complete longitudinal smartphone‐ and web‐based cognitive assessments,^85^ with this data then fed back into the DPUK data‐sharing platform to facilitate recruitment. Ongoing work by the Alzheimer's Research UK EDoN initiative goes one step further by encouraging high‐value cohorts to adopt a unified approach to prospective, digital data collection^52^ facilitating a ML approach to early detection and prognosis. Specifically, EDoN uses digital and physiological data in existing cohorts to identify factors which, via ML, develop “fingerprint models” to detect the presence of distinct dementia‐causing diseases.

### 5.2 What analytic innovations and new methods are needed?

#### 5.2.1 Novel assessment

To fully exploit the potential of remote digital cognitive assessment, the field may move beyond the abundant digital adaptation of in‐clinic neuropsychological tests. Technology can be developed to target cognitive processes anticipated to provide an early, pathology‐specific signal in an ecologically relevant context. For example, there is increasing study on the use of virtual reality to probe spatial navigation,^31^ as well as cognitive training.^86^ Altoida have produced an augmented reality object‐location task, with early evidence suggesting this tool can discriminate MCI,^87^ and an ongoing collaboration with the Global Brain Health Institute set to collect longitudinal data from 10,000 individuals on this task.^88^ In addition, data from sensor streams (e.g., microphone, touch screen, accelerometer) can be collected alongside cognitive task data to give more nuanced, in‐depth measurement. Critically, tools must be accessible for scalable adoption in terms of cost, ease of implementation, and use.

#### 5.2.2 Use of naturalistic, objective measurement

Unlike a traditional health‐care model in which help or advice is sought responsively, technological advancements in smart/wearable devices enables externally valid health associated outcomes to be tracked continuously for prolonged periods.^89^, ^90^ This can promote inclusion of under‐served communities who may have restricted access to research and health care. Devices such as accelerometers have successfully been used to classify distinct disease phenotypes, predict falls, or even prodromal disease in the field of movement disorders.^91^ Only a standardized approach will enable longitudinal and internationally comparable datasets applicable to AI and deep learning models that are well suited for prediction of cognitive decline and classifying subgroups. The shortcomings of movement disorder cohorts such as sample sizes, the lack of confirmation on dependent variables, and lack of standardized methodologies are important to overcome.^56^ Currently, those who purchase devices to monitor their personal health often are potentially more likely to be motivated and less deprived; use of digital devices may also be limited by digital illiteracy, cost, and lack of resources (e.g., no access to smartphone technology). A digitally inclusive approach is therefore key, including multiple perspectives. There are international examples: the Healthy Brain Project (Australia) uses online assessment to estimate the earliest signs of cognitive impairment.^92^ Work is required to provide affordable, unobtrusive devices to the broader population and for this information to be integrated into clinical practice and for research purposes.^93^ If the potential of smart/wearable devices are to be realized, the work required to develop standardized approaches with valid and reliable devices implementable to routine health care, should be the primary objective. A difficulty associated with use of commercial devices may be problems in data sharing at the individual level, and the ethical implications that may have including other commercial uses (e.g., health insurance), which could easily vary by government and locale. The combined innovative medicine approaches (IMI), creating international cohorts of researchers working together, appears to be a promising route.^94^

#### 5.2.3 Integration of multiple measurements with applied modeling

It is important to consider whether objective passive measurements can contribute to the detection, discrimination, and monitoring of dementia subtypes. Metrics easily collected by the same, widely adopted device (e.g., a smartphone) hold significant promise, for example, typing speed^95^ or device‐led social interactions.^96^ Much previous research uses a single digital signature to discriminate between individuals with a dementia diagnosis and healthy controls. Early detection and the discrimination of dementias with distinct, often overlapping pathologies requires a constellation of digital and low‐burden clinical variables to be analyzed together. ML methods can be applied to determine which devices provide best discrimination, considering a necessary balance between scalable, accessible assessment and predictive power. To address this, there is a need for data science that tackles how to align data collected at diverse temporal frequencies, is robust to data irregularities (e.g., introduced by software updates of bugs), and missing data.^97^ In addition, analytic techniques can be used to improve the data already in existence. For example, tools are being developed which use NLP to extract key information from unstructured, non‐uniform medical records.^80^ This will promote more widespread use of information on clinical history, drug adherence, and treatment response.

#### 5.2.4 “Bench to bedside”: Integrating modeling with real‐world benefit

Innovations in digital health and data science must be integrated in everyday healthcare to achieve real benefit. At present, there is a wealth of “proof‐of‐concept” studies evidencing the value of these tools for dementia diagnosis, prognosis, and monitoring, yet clinical practice remains largely unchanged.^98^ Indeed, myriad small validation studies, each of which explores a different digital device, trial endpoint, or statistical approach, may contribute to the lack of health‐care integration. There are several initiatives focusing on real‐world integration. The Brain Health Centre (Oxford, UK) invites participants to complete a specialist assessment (physical, remote digital, cognitive, and brain imaging) upon being referred to NHS memory assessment services. These data are processed and combined with EHRs to inform clinician decision making; consideration of novel techniques alongside patient records will transform future clinical practice although a limitation of this approach is that the “black‐box” nature of modeling can potentially harm decision‐making trust at the individual level.^99^ This issue of trust is complex: models have different stakeholders with different priorities and aim for overall (average) precision, whereas individuals may be concerned that models do not take (their) individual differences into account.^100^

Large‐scale studies and associated “big data” processing pipelines are needed to formalize which tools and measurement devices hold most promise for practical, clinical application.^101^ Pathways for regulatory approval must be clarified and adapted—greater flexibility is needed as technology and data science evolve rapidly and with burgeoning implications for personal and professional ethics. This can be facilitated by considering the distinct set of risks posed by innovation in this field, including data privacy; validation of outcomes; and effective, supported communication of risk, rather than physical safety.^102^ Engagement among clinicians, lay public, technology providers, and scientists is critical, however, to drive health‐care integration forward. A potential benefit to the field could be moving away from “black box” AI toward open‐data, transparent algorithms, and clinically relevant endpoints.^103^ In addition, solutions must be practical to implement at scale, in terms of cost, time burden, and clinician and patient/participant acceptability.

#### 5.2.5 Citizen science in digital health

Citizen science can accelerate the real‐world impact of digital and applied research by enabling rapid data collection from large, diverse populations. For example, Sea Hero Quest has used “big data” collected through citizen science (*n* = 27,108) as a benchmark for further work investigating the link between genetic and brain‐based biomarkers for AD and spatial navigation deficits.^104^ GameChanger, a study supported by the Alzheimer's Society, extends this by demonstrating the feasibility of frequent, continuous, longitudinal digital phenotyping 5 minutes a day for 30 days on an annual basis, in almost 20,000 adults aged 18 to 92 years recruited from a community setting.^105^ This establishes digital methods as a new avenue for routine population‐level, long‐term cognitive screening, which may significantly benefit early detection of dementia and diagnosis management. Before such changes can be implemented, however, we need to understand how to give meaningful individual feedback, plus whether and how more knowledge can empower and benefit patients.^106^

## 6 SUMMARY OF AI LIMITATIONS

This review has highlighted a range of limitations in applied modeling regarding health care. Several problems and limitations are fundamental and not limited specifically to one of prediction, detection, prognosis, or measurement of decline. These include (but are not limited to), first, accountability, for which the “black‐box” nature of AI/ML is such that clinical trust—among clinician, patient, and algorithm—and responsibility for decision making is complex.^106^ There are issues related to population generalizability and data equity: algorithms trained on one dataset with potential biases (sample characteristics, ancestries, biases, age ranges, etc.) may not translate effectively to other more diverse populations,^103^ for example, China Aging and Neurodegenerative Initiative^107^ and China Kadoorie Biobank cohorts.^108^ At the population level there are issues around interpretability—why have some risk factors/features been highlighted, and are those variables necessarily causal?^28^

This issue of causality is to some extent foundational in that a number of risk factors identified are either part of a generalized “protective lifestyle” (e.g., smokers are perhaps less likely to exercise), and/or may reflect part of the disease process (e.g., changes in body mass index close to dementia diagnosis).^109^ There are certain relatively fundamental obstacles in ML applied to dementia as it stands. Relatively small samples (particularly so when split into training/test); covariance among risk factors, for example, that genetic risk for dementia influences modifiable risk factors,^110^ or that lifestyle factors often inter‐correlate ‐ challenging the idea of isolated causality); and potentially only marginal gains over‐and‐above established risk factors like age, sex, and *APOE* genotype. These issues are compounded by participation and attrition bias, including with (statistically) significant impact upon exposure/outcome estimates^17^ These can to some extent be mitigated by participation “weights”,^111^ which may lead to substantially improved estimates. In the context of such limitations, it is understandable that using modeling at the individual level for clinical purposes would have to earn trust.

## 7 SUMMARY OF RECOMMENDATIONS

There is substantial promise in the use of applied modeling and digital health in dementia research going forward. Current limitations exist^112^ in terms of transparent, fast data access and the measurement information within such data and cohorts. Applied modeling and digital health in tandem have substantial promise to enhance our ability to measure risk, progression, and potential treatment response generally. In the process of that ongoing development, key themes emerge as important:

1. Emphasis on transparent, accessible, and curated data sharing including anonymized EHRs (e.g., ADDI, DPUK, DP Australia).
2. As variety of phenotyping increases (e.g., raw accelerometry, biomarkers, EHRs), the role of ML in feature decomposition and reducing large numbers of variables to key (causal) predictors, will become more critical.
3. Movement beyond existing general population/primary cohorts to secondary care outcomes, including the prediction of conversion and treatment response in clinical cohorts.
4. ML has clearly demonstrated utility in imaging in particular, but integration of larger numbers of phenotypes in prediction modeling, including, for example, the use of NLP in EHRs.
5. Digital datatypes including remote cognitive testing hold substantial promise to reduce bias in attending assessment. Incorporating more naturalistic data (e.g., accelerometers, pedometers), and developing bespoke objective data types (e.g., gait and balance) to measure physical decline, will improve modeling capability.

## 8 CONCLUSION

Improvements in applied modeling will benefit synergistically from growth and development in digital health. As the development of key applied modeling continues (e.g., in feature decomposition and integration of wider, larger data sources), and includes novel objective, naturalistic data from remote testing, data‐based prediction of dementia may become a reality.

## AUTHOR CONTRIBUTIONS

Structure and design: Donald M. Lyall, Janice M. Ranson, David J. Llewellyn, Organization: Donald M. Lyall, Andrey Kormilitzin, Team management: Andrey Kormilitzin, Claire Lancaster, Fanny Petermann‐Rocha, Jose Sousa.

Content: all co‐authors.

## CONFLICT OF INTEREST STATEMENT

Dr Kormilitzin declares research grant funding from GlaxoSmithKline. The views expressed are those of the authors and not necessarily those of the UK National Health Service, the NIHR or the UK Department of Health. All other co‐authors have no conflicts of interest to disclose.disclosures are available in the supporting information.

## CONSENT STATEMENT

Consent was not necessary or applicable.

## Supporting information

Supporting Information
